# The Effect of the Virtual Reality–Based Biofeedback Intervention DEEP on Stress, Emotional Tension, and Anger in Forensic Psychiatric Inpatients: Mixed Methods Single-Case Experimental Design

**DOI:** 10.2196/65206

**Published:** 2025-02-12

**Authors:** Lisa Klein Haneveld, Tessa Dekkers, Yvonne H A Bouman, Hanneke Scholten, Joanneke Weerdmeester, Saskia M Kelders, Hanneke Kip

**Affiliations:** 1 Centre for eHealth and Wellbeing Research Department of Psychology, Health and Technology University of Twente Enschede The Netherlands; 2 Department of Research Transfore Deventer The Netherlands; 3 Department of Child and Adolescent Psychiatry and Psychology Erasmus Medical Centre Rotterdam The Netherlands; 4 Research Group Creative Making Processes & Technology HKU University of the Arts Utrecht The Netherlands

**Keywords:** virtual reality, VR, diaphragmatic breathing, biofeedback, DEEP, forensic psychiatry, mental health, stress, single-case experimental design, experience sampling method

## Abstract

**Background:**

Decreasing aggression through stress reduction is an important part of forensic psychiatric treatment. DEEP is an experience-based virtual reality intervention that uses biofeedback to train diaphragmatic breathing and increase relaxation. Although DEEP has shown promising results in reducing stress and anxiety in students and adolescents in special education, it has not been examined in forensic psychiatric populations.

**Objective:**

This study aimed to evaluate DEEP’s potential to reduce stress, emotional tension, and anger in forensic psychiatric inpatients.

**Methods:**

A mixed methods, alternating treatment, single-case experimental design was conducted with 6 Dutch forensic inpatients. For 20 days, participants engaged in 4 DEEP sessions. Experience sampling was used for continuous monitoring of stress, emotional tension, and anger twice daily. A repeated linear mixed model was used as a primary statistical approach for analyzing the experience sampling data as well as visual analyses. Finally, semistructured interviews were conducted with participants and health care professionals to compare quantitative with qualitative results.

**Results:**

Of the 6 participants, 3 (50%) completed all 4 DEEP sessions, while the other 3 (50%) missed one session due to technical difficulties or absence from the inpatient clinic. P1 showed a significant reduction of stress after session 2 (β=–.865; *P*=.005). No significant changes over time were found, although an experienced effect was reported during the interviews. P2 showed no significant results. They reported the sessions as being repetitive, with no experienced effect. P3 showed a momentary increase of emotional tension after the first session (β=–.053; *P*=.002), but no changes were observed over time. No experienced effects were reported in the interview. P4 did not show significant results over time, and was hesitant to report clear experienced effects. P5 showed a significant decline of emotional tension (β=–.012; *P*=.006), stress (β=–.014; *P*=.007), and anger (β=–.007; *P*=.02) over time. They also reported short-term experienced effects in the interview. P6 showed a significant decline of stress over time (β=–.029; *P*<.001) and reported experiencing substantial effects. Finally, health care professionals reported a relaxing effect of DEEP in their patients but did not expect many long-term effects because no clear behavioral changes were observed.

**Conclusions:**

DEEP shows promise in teaching deep breathing techniques to forensic psychiatric inpatients, potentially decreasing stress, emotional tension, and anger in some patients. However, DEEP is not a one-size-fits-all intervention that supports every patient because the effectiveness on the outcome measures varied among participants. To increase effectiveness, emphasis should be put on supporting patients to transfer deep breathing skills into their daily lives. This highlights the importance for the structural integration of DEEP into current treatment protocols.

## Introduction

### Background

Forensic mental health care is focused on the treatment of individuals who exhibit imminent aggressive or sexually inappropriate behavior due to 1 or more psychiatric illnesses [1,2]. Reducing aggression in treatment is complex because patients often show difficulties with reflection, reading, and writing due to low literacy and treatment motivation [3,4]. However, treatment is mostly focused on talking and thinking about behavior, guided by treatment frameworks such as cognitive behavioral therapy, with less attention for more experience-based or experimental approaches [5]. This shows a need for interventions that can support patients in preventing aggressive behavior in an experiential and engaging way.

Earlier research has shown the promise of virtual reality (VR) and serious games to better involve patients with lower cognitive skills and treatment motivation [5,6]. Previous research has also shown that the VR game DEEP fits the skills and needs of the forensic population [7]. In DEEP, the player moves through an immersive world where they have to use deep diaphragmatic breathing to navigate through and explore the world [8]. The user receives feedback on how they are breathing, which helps them to adjust their breathing and learn to apply diaphragmatic breathing. This technique of providing direct feedback on bodily signals is called biofeedback [9]. Diaphragmatic breathing has been shown to support relaxation, well-being, and anger regulation [10,11].

Previous research has shown the potential of DEEP to reduce negative emotions. First, a randomized controlled trial with DEEP in undergraduate students showed a decrease in anxiety symptoms after using DEEP 4 times [12]. However, these results cannot be generalized, as the study population consists of a relatively healthy, highly educated, and homogeneous target group, which is not the case for the forensic psychiatric population. Second, a single-case experimental design (SCED) study with adolescents in special education demonstrated DEEP’s ability to decrease state anxiety and stress [11]. By using an SCED, this study was able to provide insights into both short-term and potential learning effects of DEEP. Moreover, their level of disruptive behavior in class seemed to decrease [11]. Although this population and the forensic target group have overlapping characteristics, the results are not entirely generalizable to a forensic setting. Namely, this study did not include state anger or aggression, which are important treatment outcomes in forensic mental health. There seems to be a need for more specific studies that investigate whether DEEP is fitting the characteristics of the forensic psychiatric setting, focusing on those specific outcomes measures. On the basis of these earlier studies and qualitative research in which DEEP was discussed with forensic patients and health care professionals, DEEP could be a fitting intervention for the hard-to-involve forensic psychiatric patient group. First, because of its appealing and immersive design in VR, DEEP might be able to engage populations with lower motivation, which is not always possible with regular breathing or relaxation interventions [7]. Second, DEEP uses diaphragmatic breathing, where an individual learns to breathe deeply using the diaphragm muscle, as opposed to shallowly through the rib cage and chest [13-15]. In addition to direct relaxation, the use of deep breathing during stressful situations can enhance emotion regulation and reduce stress [16]. By reducing stress, applying deep breathing may also reduce anger and aggression, because these constructs are important factors that may underlie outbursts of aggression [17-19]. By letting patients experience and adjust their own way of breathing in an engaging way, DEEP might be able to support them in increasing relaxation and well-being and decrease emotional tension, stress, and anger.

### Objectives

This study aimed to investigate whether DEEP can support forensic inpatients in reducing stress, emotional tension, and anger.

In this mixed methods SCED study, the following research questions were formulated:

1. To what extent is there a significant improvement in self-reported stress, emotional tension, and anger in forensic psychiatric inpatients on days when DEEP is being used compared to days when it is not used?
2. To what extent is there a significant improvement in self-reported stress, emotional tension, and anger in forensic inpatients over time during the period when DEEP was used?
3. To what extent do patients and health care professionals observe or experience effects on emotional tension, stress, and anger that they attribute to DEEP?

## Methods

### Study Design

To answer the research questions, a mixed methods SCED was applied. Data on stress, emotional tension, and anger were collected quantitatively via experience sampling (ES) as well as qualitatively via interviews. In SCEDs, participants serve as both the intervention group and their own control group. Only a small number of participants are needed to provide sufficient statistical power (3-6), with a minimum of 3 data points per phase, including a baseline and follow-up [20]. Therefore, an SCED is a fitting study design to use within the forensic psychiatric population, as they form a heterogeneous and complex-to-involve target group with high comorbidity. To study the effectiveness of DEEP, an alternating treatment SCED was chosen [20]. In this type of design, an intervention is not applied on certain days (A phase) and is implemented on other days (B phase), which is then repeated often in a brief period [21,22].

### Setting and Participants

The study was conducted at Dutch inpatient clinics with various security levels. Five health care professionals (ie professionals providing social and behavioral support in mental health settings) were included. Of these, 4 (80%) identified as female and 1 (20%) as male, with ages ranging between 27 and 39 (mean 31.2, SD 7.1) years. Patients were included via convenience sampling, which meant that they were included via their health care professional, who identified them as suitable candidates and asked them to participate. A patient was included if (1) they were aged ≥18 years, (2) they were admitted to a forensic psychiatric hospital, (3) they were expected to reside in the same clinic for at least 5 weeks, (4) they were treated for aggression regulation problems, and (5) their health care professional deemed them physically and mentally capable to participate. A patient was excluded if (1) they experienced psychotic episodes or any other form of psychiatric crisis that could negatively impact participation, or (2) they experienced epileptic episodes. In total, 6 forensic inpatients were included. All (6/6, 100%) identified as male, with ages ranging between 23 and 45 (mean 33.4, SD 9.3) years. The data collection commenced 1 week after inclusion, allowing patients sufficient time to reconsider and withdraw from the study if they wished. The inclusion of participants was done in line with the guidelines from the Declaration of Helsinki and from the Medical Research Involving Human Subjects Act (wet medisch-wetenschappelijk onderzoek met mensen in Dutch).

### Materials

#### Avicenna App

During the SCED, the participants were monitored through ES, providing insight into outcome measures, which were measured multiple times per day [23]. ES increases the ecological validity, possibilities, and reliability of frequent data collection [24]. The participants received questions regarding their experienced stress, emotional tension, and anger by use of the Ethica app (formerly known as the Avicenna app), which assists researchers in collecting real-time data [25]. During the study period, participants received 3 notifications per day (at 10:00 AM, 2:00 PM, and 9:00 PM) through the installed app on their mobile phones with a set of 3 statements about the study outcomes: “At this moment I feel [emotionally tense, stressed or angry].” The participants scored these statements on a 5-point Likert scale, where 1 was “not the case at all” and 5 was “very much the case.”

#### The DEEP Intervention

**Figure 1:**
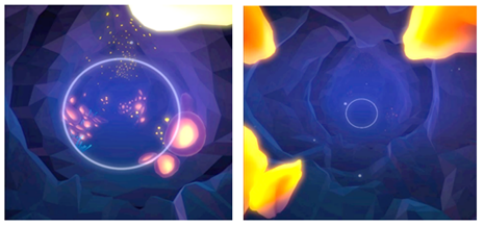
A visual representation of a deep breathing exercise in DEEP, as measured with an abdominal belt, with the left image showing a breathing circle during the measured inhalation and right during exhalation.

DEEP is a VR game based on scientific knowledge about anxiety and stress regulation [8]. The player finds themselves in an immersive virtual underwater world where they navigate using their diaphragmatic breathing, which is measured by an abdominal belt [8,26]. The player’s breathing is visualized in the VR environment through a breathing circle, as well as by the illumination and growth of plants and corals, which mirror the player’s breath (Figure 1). By using deep breathing, the player is able to increase their progress in the game, while shallow breathing, which often occurs while experiencing negative emotions, will lead to in-game stagnation. During this study, a beta-version of DEEP was used on the Oculus Rift headset. Oculus (currently known as Meta Quest) is a well-known provider of VR technology and is Conformité Européene certified [27]. In this study, the participants would sit on a spinning desk chair with the VR headset on their head and the abdominal belt around their diaphragm. Both the headset and the belt were attached to a laptop by wires.

### Procedure

#### Informing and Including Participants

First, the health care professional would ask the patient for approval to be approached by the researchers and to provide them a flyer and information letter. If the patient expressed interest in participating, an appointment was scheduled with the patient to discuss the study. During this meeting, the researcher informed the patient about the time commitment and measurement methods. The health care professional was not present at this meeting to avoid role conflicts that could potentially hinder the voluntary decision to participate due to the dependency relationship with their health care professional. If the patient felt adequately informed and decided to participate in the study, they signed an informed consent. Next, the researcher helped the patient in installing the Avicenna app and assisted them in completing the first set of questions regarding stress, emotional tension, and anger on Avicenna, for instance, by reading them aloud.

#### Study Procedure

The SCED was conducted between May 2022 and October 2022 by one researcher (LKH). An overview of the SCED procedure is provided in Table 1.

**Table 1:** Overview of the alternating treatment design conducted with forensic inpatients from a Dutch forensic psychiatric organization (N=6).

	3 days	1 day	3 days	1 day	3 days	1 day	3 days	1 day	3 days
P1	Baseline (A)	DEEP (B)	No DEEP (A)	DEEP (B)	No DEEP (A)	DEEP (B)	No DEEP (A)	DEEP (B)	No DEEP (A)
P2	Baseline (A)	DEEP (B)	No DEEP (A)	DEEP (B)	No DEEP (A)	DEEP (B)	No DEEP (A)	DEEP (B)	No DEEP (A)
P3	Baseline (A)	DEEP (B)	No DEEP (A)	DEEP (B)	No DEEP (A)	DEEP (B)	No DEEP (A)	DEEP (B)	No DEEP (A)

In the B phase, DEEP was introduced to the participant. During a period of 20 days, DEEP was introduced 4 times, based on a previous study on DEEP [11]. The researcher visited the location to prepare the DEEP session and supported the patient in its use. DEEP was started via the laptop, which meant that the researcher could watch along with what the participant was seeing through the headset. Before the first session, the researcher provided participants with a brief explanation of the DEEP concept, deep breathing, and what they would be doing during the DEEP sessions. During the first session, the researcher also provided some help if a participant would get stuck or felt lost in the game. The other 3 sessions were done by the patient independently, while the researcher was there to observe.

#### Semistructured Interviews

A week after the completion of the SCED, 2 separate semistructured interviews (mean 38, SD 4.23 min) were conducted with participating patients and their daily health care professionals to assess whether they experienced an effect of DEEP on the level of stress, emotional tension, and anger of the participant and if any other benefits or disadvantages for the participants and their behavior had been observed. This was further expanded by asking the participants whether DEEP had an effect during the session, the day of the session, and in their daily lives in general. The health care professionals were asked the same types of questions about the patient’s experienced stress, emotional tension, and anger, and they were also asked about the behavior of the participant during the SCED.

### Data Analysis

#### Visual Analyses

The responses to the ES questions resulted in a maximum of 60 data points per participant (3 times per day for 20 days). Time series graphs were constructed per participant in R (version 4.0.0; R Foundation for Statistical Computing) using the *scplot* package (version 0.3.8), which is an add-on package to the *scan* package (version 0.60.0) for visualizing single-case data [28]. Each graph illustrated stress, emotional tension, and anger over time, with days on which DEEP was used marked. Visual analysis was primarily used to gain an overall impression of the data [29,30]. Of the 6 aspects of visual analysis to consider in single case research [29], the level, variability, and trend of each phase were inspected. Level refers to the difference in mean values between phases. Variability refers to the fluctuation of the data, in this case reflected by the data’s range. Trend refers to the slope of the best-fitting line for data within a phase and is used to examine whether the outcomes decreased or increased over time and whether the introduction of the intervention changed this trajectory [29].

#### Repeated Linear Mixed Model Analysis

The data were statistically analyzed using linear mixed models (LMMs) embedded in the MultiSCED web application [31]. MultiSCED is an interactive web application that provides R functionality. LMMs compute 4 parameters—intercept, time effect, intervention effect, and interaction of intervention over time—for each participant, enabling phase-wise comparisons at the participant level. Using MultiSCED, individual linear models were constructed for each participant to determine the individual effects of DEEP on stress, emotional tension, and anger. The additional use of SCED-specific fit indices to statistically examine the nonoverlap between data points across phases A and B such as nonoverlap of all pairs [32], treatment as usual–uncontrolled [33], and typicality of level change [34] were considered. However, this approach was abandoned as half of the participants did not reach the sufficient number of 5 data points in the B phase required for reliable effect sizes [34].

#### Semistructured Interviews

A deductive approach was used to analyze the interview data [35]. This way, potential findings from both the ES method (ESM) data and interviews complement each other. The interviews were recorded and transcribed verbatim. Afterward, the transcriptions were coded deductively for the outcome measures stress, emotional tension, and anger. Any relevant fragments regarding other outcomes measured were inductively coded. On the basis of these fragments, an initial version of the coding scheme with main code and subcodes was established [36]. This coding scheme was then independently applied to the first 3 interviews by the first coder (LKH), which then was sent to the second coder (HK). An agreement rate of 81% was found, which is acceptable in the coding process [37].

### Ethical Considerations

The Single-Case Reporting Guideline in Behavioral Interventions (SCRIBE) was used to design and report this study [38]. The checklist can be found in Multimedia Appendix 1 [38]. Ethics approval was given by the Ethics Committee of the University of Twente (Behavioral, Management and Social Sciences, no. 220190). Each patient was thoroughly informed about the study through information folders, information letters, and clarification from the researcher. Before participating, every patient signed an informed consent form. As compensation for their participation, all patients received 2 vouchers worth €10 (US $10) halfway through and at the end of the study. To ensure privacy and confidentiality, personal data regarding age, gender, and psychiatric disorder were stored on the secured research drive of the University of Twente. Moreover, names of patients and therapists were omitted from the transcripts, and only participant codes were used to connect quantitative and qualitative data, resulting in a pseudoanonymized dataset.

## Results

### Overview

The results of the ES and interviews are reported for each participant individually. The interview results per participant are reported using the codes regarding the outcomes measures, that is, stress, emotional tension, and anger. The coding schemes on group level can be found in Multimedia Appendix 1.

### Descriptive Statistics

The mean scores on stress, emotional tension, and anger per phase are depicted in Table 2. Half of the participants (3/6, 50%) showed lower average levels of emotional tension (P4-P6), and most lower levels of anger (P1 and P4-P6) and stress (P1 and P3-P6). However, other participants reported more stress (P2), emotional tension (P2 and P3), and anger (P2) on DEEP days.

**Table 2:** Descriptive statistics of experience sampling data on emotional tension (ET), stress, and anger per participant per phase (scored with a 1-5–point Likert scale).

Participant and outcome			Reported range in Likert scale (1-5)		A phase, mean (SD)		B phase, mean (SD)		Total missing datapoints, for scoring stress, anger and ET combined, n (%)
**P1**									
	Stress	3-5		4.00 (0.24)		3.75 (0.5)		—^a^	
	ET	3-4		3.92 (0.28)		4 (0)		20 (33)	
	Anger	2-4		3.39 (0.60)		3 (0.82)		—	
**P2**									
	Stress	2-5		2.28 (0.75)		3 (1.41)		—	
	ET	2-5		2.30 (0.76)		3 (1.41)		12 (20)	
	Anger	1-5		2.39 (0.93)		3 (1.41)		—	
**P3**									
	Stress	2-5		2.08 (0.49)		2 (0)		—	
	ET	1-4		2.03 (0.37)		2.5 (1)		18 (30)	
	Anger	2-4		2.05 (0.32)		2 (0)		—	
**P4**									
	Stress	2-4		2.12 (0.45)		2 (0)		—	
	ET	2-4		2.26 (0.66)		2 (0)		15 (25)	
	Anger	2-5		2.23 (0.68)		2 (0)		—	
**P5**									
	Stress	2-4		2.59 (0.69)		2.50 (0.71)		—	
	ET	2-4		2.42 (0.60)		2 (0)		4 (7)	
	Anger	1-3		2.13 (0.39)		2 (0)		—	
**P6**									
	Stress	1-4		2.64 (1.01)		2.25 (1.26)		—	
	ET	1-4		2.98 (0.93)		2.25 (1.26)		1 (2)	
	Anger	1-5		1.82 (1.14)		1.50 (0.58)		—	

^a^Not applicable.

### Results of ES and Interviews

The results of the visual analyses, LMMs, and interviews are described per participant. At the end of this section, a synthesis of the qualitative and quantitative results is presented. An overview of the LMM outcomes can be found in Multimedia Appendix 1.

#### Results for P1

**Figure 2:**
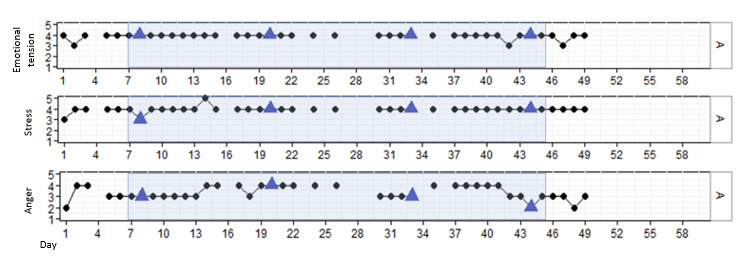
Stress, emotional tension, and anger in P1 over time, measured with experience sampling. The light blue marking represents the period DEEP was introduced and withdrawn. The blue triangles indicate the first data point after a DEEP session.

P1 was a man aged 44 years who, according to himself, showed difficulty controlling his temper when others irritated or did not agree with him. During the study, P1 was enthusiastic about working with DEEP and was interested in how breathing in a VR environment would help him regulate his emotions. P1 completed all 4 DEEP sessions with no difficulty. Figure 2 illustrates that P1 showed consistent levels of high emotional tension and stress independent of DEEP use. In addition, a significant momentary reduction in stress was found after session 2 (β=–.865; *P*=.005). However, this effect disappeared over time (β=.023; *P*=.02). Although no significant changes over time were found, anger (β=–.001; *P*=.93) seemed to variate more than stress (β=–.002; *P*=.57) or emotional tension (β=–.002; *P*=.52).

The interview outcomes and examples of quotes regarding those outcomes are shown in Table 3. During the interview, P1 indicated that he found DEEP a pleasant experience. P1 felt that DEEP was more of a helpful reference to understand deep breathing than a direct intervention in increasing emotion regulation. His advice was to not only offer DEEP as a stand-alone intervention but also provide patients with assignments, which can help to better apply deep breathing into daily life situations. Because P1 was trying out for assisted living, he had little contact with health care professionals during this study. Therefore, his health care professionals had no clear view on his daily emotions and behavior during this study.

**Table 3:** Results of the semistructured interview with P1 regarding the experienced effects on emotional tension, stress, and anger.

Outcome measure	Times mentioned	Example quotes from P1
Emotional tension	5	“DEEP became a reference for me. I do the session and think back on it. Like oh, I feel tense, let’s pretend that I am doing DEEP. Just in my head, feeling like a little fish.”
Stress	2	“And I’ve actually had quite a bit of trouble with panic attacks and stress. But it’s all pretty much gone now. And when I start feeling a bit off, I just focus on my breathing.”
Anger	1	“And it helped just as well with anger. Absolutely. Simply by breathing. Just going back. Back to basics, back to swimming and just breathing calmly.”

#### Results for P2

**Figure 3:**
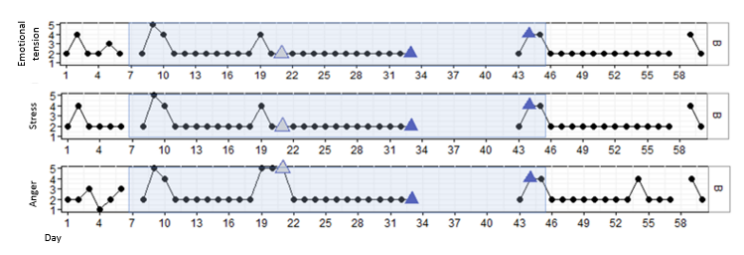
Stress, emotional tension, and anger in P2 over time, measured with experience sampling. The light blue marking represents the period DEEP was introduced and withdrawn. The blue triangles indicate the first data point after a DEEP session. The gray triangles indicate a DEEP session in which no measurements were taken.

P2 was a male patient aged 23 years who, according to his health care professionals, was enthusiastic about his treatment but also impulsive and manipulative in nature. He was glad to participate in this study and was curious to find out whether the deep breathing skills he learned in therapy could support him during a DEEP session. P2 completed 3 out of 4 DEEP sessions. The first session was canceled due to a conflict between him and another inpatient. Figure 3 shows that P2 reported some variation in stress, emotional tension, and anger at the start of the study, which stabilized after data point 10 (emotional tension and stress) and data point 22 (anger). In addition, all outcomes temporarily increased after his last use of DEEP. However, no significant results in stress (β=.002; *P*=.57), emotional tension (β=–.006; *P*=.29), or anger (β=–.002; *P*=.78) were found over time or directly after DEEP sessions.

During the interview, P2 indicated that he really liked the first DEEP session but that the last session felt repetitive as he was doing the same thing each time. Moreover, he found the DEEP sessions relaxing, but did not find it to be helpful during moments of conflict. The health care professional of P2 did not really notice any changes in behavior after the DEEP sessions. She did acknowledge that it might have had a positive effect on his well-being, as he was more aware of his breathing. However, during a conflict, she did not notice that he was less angry or that he was able to regulate his emotions through deep breathing (Table 4).

**Table 4:** Results of the semistructured interview with P2 regarding the experienced effects on emotional tension, stress, and anger.

Outcome measure	Times mentioned	Example quotes from P2
Emotional tension	5	“I would recommend DEEP to other patients. Not everyone would maybe benefit, but it could help most of them with developing breathing skills and a better way to relax and become less tense.”
Stress	1	“I did find the DEEP sessions to be effective in reducing my stress. Because I found that when I really focused on my breathing I became more calm and mindful.”
Anger	1	“But I do notice that with DEEP, whenever I get stressed or angry or things like that, I do pay attention to my breathing. I don’t realize it. It’s already ingrained in my system [due to earlier treatment that used breathing exercises] that I do that.”

#### Results for P3

**Figure 4:**
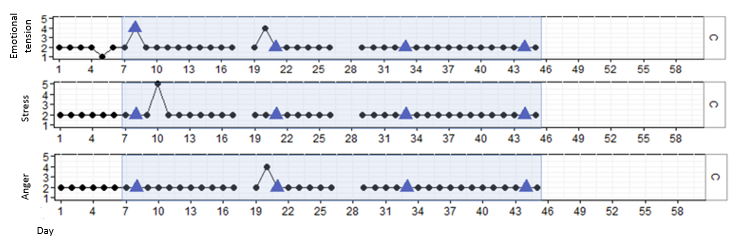
Stress, emotional tension, and anger in P3 over time, measured with experience sampling. The light blue marking represents the period DEEP was introduced and withdrawn. The blue triangles indicate the first data point after a DEEP session.

P3 was a man aged 31 years. His health care professionals indicated that he sometimes struggles to access his emotions and talk about them. P3 was interested in participating because he has worked in the IT field and was intrigued by the technology behind the VR game and how the biofeedback in DEEP operates. P3 completed all 4 DEEP sessions. Figure 4 shows that P3 reported low levels and little fluctuation of stress, emotional tension, and anger throughout the study. In addition, his reported emotional tension was significantly increased after the first session (β=1.875; *P<*.001). However, this effect did not remain over time (β=–.053; *P*=.002), indicating only a momentary increase in tension with first-time use of DEEP. Finally, no changes over time in stress (β=–.006; *P*=.34), emotional tension (β=.002; *P=*.71), and anger (β=–.001; *P*=.85) were found.

During the interview, P3 stated that he expected more from the DEEP sessions. He felt that the VR showed some technical defects and that it did not help him in reducing negative emotions. According to his health care professional, P3 was not entirely engaged in DEEP and what it might teach him. He was more involved in the technical part, such as the VR and abdominal belt, and expressed annoyance that it did not always work correctly (Table 5).

**Table 5:** Results of the semistructured interview with P3 regarding the experienced effects on emotional tension, stress, and anger.

Outcome measure	Times mentioned	Example quotes from P3
Emotional tension	2	“I was able to focus on my breathing during DEEP, which made me feel relaxed. But I already knew this from going to the gym and deep breath during my exercises.”
Stress	1	“I don’t know if DEEP helps with reducing stress. I would say that you would need more triggers to test that. Like that things get a bit scary in the game and that you have to keep breathing to calm yourself down.”
Anger	2	“Something just came to mind. I don’t even remember what it was about, but I was irritated recently. But expressed it calmly, and was immediately vocal about it, you know. But then, did I take a breath? No, that feels pointless.”

#### Results for P4

**Figure 5:**
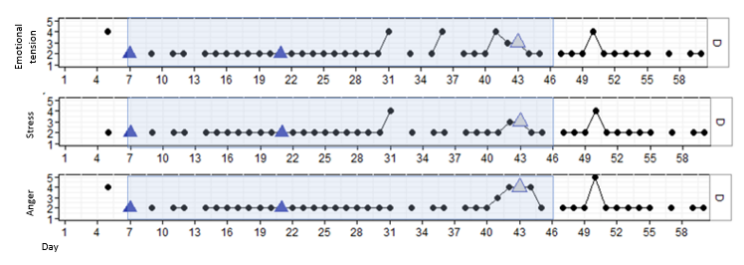
Stress, emotional tension, and anger in P4 over time, measured with experience sampling. The light blue marking represents the period DEEP was introduced and withdrawn. The blue triangles indicate the first data point after a DEEP session. The gray triangles indicate a DEEP session in which no measurements were taken.

P4 was a man aged 24 years. According to his health care professionals, he was a quiet man who had difficulty recognizing and articulating his own emotions. P4 completed 3 of 4 sessions. The third session was canceled due to the researcher being unavailable unexpectedly. As is shown in Figure 5, P4 reported low stress, emotional tension, and anger during the first half of the study. More variety in these outcomes was observed in the second half of the study. However, no significant results in stress (β=.004; *P*=.44), emotional tension (β=.000; *P=*.97), and anger (β=.004; *P*=.61) were found over time, nor was a direct effect observed after a DEEP session.

During the interview, P4 stated DEEP sessions to be a fun and relaxing experience. He also felt that after a DEEP session, he was able to stay calm the rest of the day, even when conflict occurred. However, P5 was hesitant to state that DEEP helped him using his deep breathing in daily life situations. Except for during a DEEP session, he did not use his deep breathing to cope with negative emotions, such as anger. His health care professional was also hesitant to ascribe a direct effect of DEEP on P5’s behavior. She did find P5 to be calm and tired after a DEEP session and indicated that it provided him with more insight on deep breathing. However, she did not find him less tense, stressed, or angry during the SCED, as he was already behaving well in the clinic (Table 6).

**Table 6:** Results of the semistructured interview with P4 regarding the experienced effects on emotional tension, stress, and anger.

Outcome measure	Times mentioned	Example quotes from P4
Emotional tension	6	“I loved the DEEP sessions. It was nice and really relaxing. Just to be in a calm environment made me less tense already.”
Stress	4	“DEEP gave me an empty mind. I had nothing to think or stress about. The mornings you [researcher] came around with DEEP were nice days. I could really relax.”
Anger	4	“After the session we were cooking, and there was someone bossing people around, and normally I would say: do something yourself for once! Normally, I would react with irritation, but now I was very calm and could respond more calmly.”

#### Results for P5

**Figure 6:**
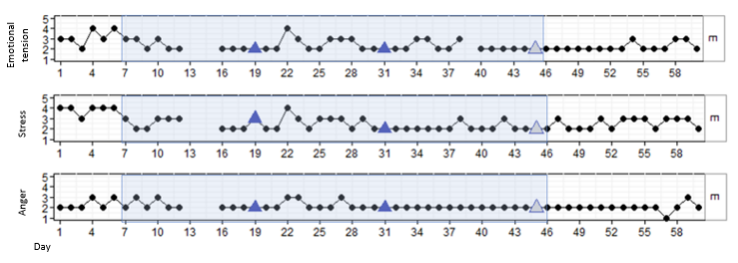
Stress, emotional tension, and anger in P5 over time, measured with experience sampling. The light blue marking represents the period DEEP was introduced and withdrawn. The blue triangles indicate the first data point after a DEEP session. The gray triangles indicate a DEEP session in which no measurements were taken.

P5 was a man aged 37 years. According to both him and his health care professional, he did not always recognize when his anger was increasing and could suddenly explode. He was curious about how DEEP could help him in recognizing his increasing emotional tension and using deep breathing techniques in a timely manner. P5 completed 3 of 4 DEEP sessions. The first session was canceled due to a technical problem with the VR set. As is shown in Figure 6, P5 reported fluctuations on the 3 outcome measures. The DEEP sessions were consistently associated with the lowest scores on emotional tension, stress, and, to a lesser extent, anger. In addition, a significant decline over time in emotional tension (β=–.012; *P*=.006), stress (β=–.014; *P*=.007), and anger (β=–.007; *P*=.02) was found, with high to moderate levels of stress until day 9 and moderate to low levels of stress after that.

During the interview, P5 stated that DEEP was a helpful intervention for him to relax and focus on his own body. However, P5 was not able to use his deep breathing techniques and reduce his anger during conflicts or stressful situations. He stated that during those moments, he is so engaged in the conflict that he is not thinking about his bodily signals or what he should do to prevent aggression. His health care professional agreed with these statements. She acknowledged that P5 really felt happy and relaxed after each DEEP session and that he was able to keep calm during most conflicts. However, the health care professional did not find DEEP to be effective in supporting P5 during stressful moments or aggressive outbursts (Table 7).

**Table 7:** Results of the semistructured interview with P5 regarding the experienced effects on emotional tension, stress, and anger

Outcome measure	Times mentioned	Example quotes from P5
Emotional tension	5	“You know, one thing I’ve become more aware of is my breathing. Even before getting out of bed, I sometimes pay attention to my abdominal breathing. So, I’m conscious that breathing can be a momentary relief of tension to start and get through the day.”
Stress	2	“I do feel less stressed, but I am not entirely sure if it can be linked to doing DEEP. I think I have to do more DEEP sessions to discover that.”
Anger	2	“During DEEP I was in a calm environment to chill and get out of my head. But I don’t know if those breathing exercises work if I am in a fight with a family member. I don’t think so to be honest.”

#### Results for P6

**Figure 7:**
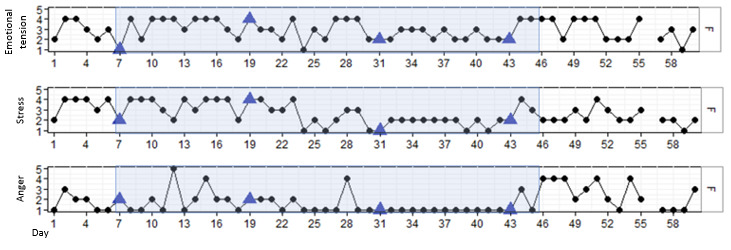
Stress, emotional tension, and anger in P6 over time, measured with experience sampling. The light blue marking represents the period DEEP was introduced and withdrawn. The blue triangles indicate the first data point after a DEEP session.

P6 was a man aged 33 years. According to his health care professional, he often struggled to regulate his negative emotions in a healthy way. DEEP appealed to P6 because he wanted to find distraction through it and calm his thoughts. P6 completed all 4 DEEP sessions. However, the second session for P6 was interrupted due to a signal disruption between the VR headset and the VR sensor after 8 minutes. The session was eventually completed but the participant did experience more discomfort than usual, because his vision kept shifting, causing difficulty to navigate properly. As is shown in Figure 7, P6 reported both highly fluctuating levels of stress, emotional tension, and anger. His findings are mixed, with DEEP sessions being associated with both the least (session 1; day 2) and highest (session 2; day 6) emotional tension. However, anger levels did seem to decrease with the use of DEEP, notably after session 3 (day 10), which was followed by a longer period of stability. In addition, a significant decline of stress was found over time (β=–.029; *P*<.001) but not in emotional tension (β=–.009; *P*=.22) or anger (β=.009; *P*=.33). In addition, as neither of the interaction effects are significant, these declines cannot be attributed directly to a momentary effect of DEEP.

During the interview, P6 expressed being pleasantly surprised by how much he liked the DEEP sessions. He found the environment and music of DEEP to be a great distraction from his negative thoughts and felt that it helped him to cope with his negative emotions. According to his health care professional, P6 really experienced that he can use his deep breathing to reduce emotional tension and stress. She stated that, although there were some conflicts during the data collection period, P6 clearly was less angry and explosive toward the health care professionals (Table 8).

**Table 8:** Results of the semistructured interview with P6 regarding the experienced effects on emotional tension, stress, and anger.

Outcome measure	Times mentioned	Example quotes from P6
Emotional tension	6	“What really stuck with me was that I started every DEEP session tense and that I came out of the session calm and happy.”
Stress	2	“I do remember that, during the DEEP sessions, I was able to breath in a deep and calm way which felt really nice and made my body relax as well.”
Anger	3	“I’ve had quite a rough couple of weeks. And after the DEEP sessions, I noticed that I didn’t immediately react to conflicts, but found a way to cope. I didn’t explode right away but managed to keep myself together. Also, because I emerged from such a session feeling so relaxed.”
Self-efficacy^a^	2	“I know that if I explode, I bring a lot of tension to the staff and the group. DEEP helped me to respond more calmly. But now the study has ended, and I don’t know how I will do without the help of DEEP.”

^a^This was not a predetermined outcome measure but additionally mentioned by P6.

### Synthesis of the Results

In this study, we used multiple research methods to assess the effectiveness of DEEP on stress, emotional tension, and anger in forensic psychiatric patients. In Table 9, we juxtaposed the quantitative and qualitative results per participant. This provides an overview on group level and an individualized examination of whether they paint a consistent picture or present contradictory results. Some discrepancies were observed between quantitative and qualitative results. Two patients showed no significant changes quantitatively, but reported experiencing effects of DEEP in the interviews. Two other patients displayed short-term significant changes in emotional tension or stress, with one patient reporting matching experiences and the other reporting no effects. The final 2 patients showed significant changes over time, aligning with their reported experiences.

**Table 9:** Overview of quantitative and qualitative results per participant (N=6).

ID	Quantitative outcomes	Qualitative outcomes
P1	Variation in stress, ET^a^, and anger Momentary decrease of stress No significant changes over time	Experienced short-term effect on ET and stress No experienced long-term effect
P2	Stabilization of stress, ET, and anger in second half SCED^b^ No significant changes over time	Experienced short-term effects on ET No experienced long-term effects
P3	Low stress, ET, and anger throughout SCED Momentary increase of ET No significant changes over time	No experienced short-term effects No experienced long-term effects
P4	More fluctuation of stress, ET, and anger in second half SCED No significant changes over time	Experienced short-term effect on ET, stress, and anger Experienced long-term effect on ET
P5	Fluctuation, but lower levels in stress, ET, and anger after DEEP sessions. Significant reduction of stress, ET, and anger over time	Experienced short-term effect on ET and stress Experienced long-term effect on ET and stress
P6	Elevated levels and fluctuation of stress, ET, and anger throughout SCED Significant reduction of stress over time	Experienced short-term effect on ET, stress, and anger Experienced long-term effect on stress and anger

^a^ET: emotional tension.

^b^SCED: single-case experimental design.

## Discussion

### Principal Findings

This study aimed to investigate whether DEEP, through its immersive focus on relaxation and diaphragmatic breathing techniques, is able to decrease stress, emotional tension, and anger in forensic psychiatric inpatients. First, much variation in effectiveness was found between each participant. In 2 participants, a momentary effect on emotional tension and stress right after a DEEP session was found. Two other participants showed a decrease in stress, emotional tension, and anger over time, with no direct effect of DEEP. The last 2 participants did not show any significant effects. These results underline that DEEP is no one-size-fits-all solution and that users might benefit from DEEP in diverse ways or not at all. Second, most participants felt enthusiastic about DEEP and found it to be a helpful intervention to enhance short-term relaxation. Some participants also found DEEP to be able to teach them diaphragmatic breathing, which, in their experience, helped them reduce aggressive outbursts or conflicts. However, most patients were not yet able to put what they had learned in DEEP into practice, which was also indicated by their health care professionals. Finally, some discrepancies were found in the synthesis of the ES and qualitative results; although most patients experienced a positive effect of DEEP on their well-being, 67% (4/6) of them showed no significant change in emotional tension, stress, or anger.

### Variation in Effectiveness

An important finding of this study is that DEEP seems able to reduce momentary emotional tension, stress, and anger and induce short-term relaxation in some but not all forensic inpatients. One possible explanation for this is that the participants in this study exhibit heterogeneity in their history of offenses, treatment objectives, cognitive abilities, and psychosocial characteristics [4,39], which might explain the differences in their experiences during the DEEP sessions and variation in effectiveness. This underlines earlier research, which states that there is no one-size-fits-all approach to support all psychiatric patients in their treatment and that a personalized approach is essential [40], which is in particular the case for forensic patients [3]. Furthermore, DEEP was provided as a stand-alone intervention within a research context without a more personalized instruction. The participants stated that they had trouble applying what they learned in DEEP into daily life situations. This shows that a structural integration of DEEP into existing treatment could support patients in generalizing deep breathing skills, making the intervention more effective as is supported by earlier studies. Finally, some technical difficulties with the abdominal waistband occurred during the study, which limited the impact biofeedback could have on participants. Future research could further investigate these reasons for variation in effectiveness, for example, by focusing on patient-, contextual-, and intervention-related factors [41-43].

### Generalization of Deep Breathing Skills

Another finding of this study is that DEEP was able to induce short-term effects on stress, emotional tension, and anger. However, a long-term effect that could directly be ascribed to DEEP was not found in any participants. Similar to earlier evaluation studies of DEEP, the DEEP sessions were conducted as a “stand-alone” intervention, without an extensive introduction or debriefing from a health care professional [8,11,26]. In the interviews, patients and health care professionals stated that more information about diaphragmatic breathing could help them to actively use this as a coping strategy to prevent aggression in their daily lives. Therefore, DEEP might require some form of psychoeducation and support with integration in daily life to generalize the skills that are taught in DEEP [7]. In addition, other studies recommended that technological interventions, such as VR, should be embedded within existing treatment as opposed to being used as a separate tool [6,44,45]. Therefore, future research should focus on determining the most optimal way to integrate DEEP in a treatment context where health care professionals are conducting the DEEP sessions.

### Using Deep Breathing During Conflicts

A third finding of this study is that most participants often forgot to apply diaphragmatic breathing during conflicts because they were already too involved or angry. An explanation for this is that patients were often not aware of their increasing emotional tension or anger, which is a common problem in forensic psychiatric patients [46]. Consequently, necessary precondition for applying diaphragmatic breathing to prevent aggression seems to be that patients recognize their increasing tension early on [18,47,48]. This degree of recognition and trust in one’s own bodily signals—which is called interoceptive awareness—is indeed found to be related to the improvement of emotion regulation skills [48]. Therefore, it would be interesting to determine whether the effect of DEEP or other emotion regulation interventions can be enhanced if the user’s interoceptive awareness increases. Consequently, a recommendation for future research is to combine DEEP with other biofeedback devices such as wearables that can be used to increase interoceptive awareness in daily life [46,47].

### Strengths and Limitations

First, by using a mixed methods single-case approach, this study was able to differentiate individual responses to a novel intervention. However, the data quality of the current SCED study limited opportunities to use SCED-specific effect sizes such as treatment as usual–uncontrolled, nonoverlap of all pairs, and typicality of level change and, as such, to be able to draw robust conclusions about the effectiveness of DEEP. To meet standards in future studies, at least 5 data points in each phase should be produced. This could be successfully reached by extending the DEEP phase by embedding the VR sessions in treatment as usual. This way more data points are easily collected and long-term changes in outcome measures might be reached. Second, by using convenience sampling as a sampling method, researchers and health care professionals were able to recruit and include participants, while ensuring a time- and cost-effective procedure [49]. However, it could have led to selection bias as health care professionals may have only recruited patients that they deemed motivated to participate to ensure that they were able to complete the study [49,50]. Therefore, it is important to remain careful when generalizing study outcomes, particularly as the vulnerable forensic psychiatric patients could not be involved in this study. Third, attention should be paid to the impact of technical issues, as some DEEP sessions were canceled or interrupted due to signal loss between the abdominal belt and the VR equipment. It should be noted that these issues could have inhibited the possible effectiveness of DEEP. Fourth, this study used ES, which is a suitable way to study real-time changes in mental states [51,52]. As a significant decrease in stress, emotional tension, and anger over time—without a direct effect of DEEP—was found in 2 patients, ES could have provided an unforeseen effect on their well-being, just by asking them to reflect on their emotions thrice a day. However, by using a mixed methods design, these possible unforeseen effects have been partly addressed in semistructured interviews because quantitative ESM data were supplemented by experienced effectiveness. Finally, emotional tension, stress, and anger are complex constructs to measure, particularly using ES [53]. Some participants mentioned effects of DEEP that were not reflected in the ESM data, raising questions about the extent to which the items were fully valid. Finally, participants scored their stress, emotional tension, and anger by using a 5-point Likert scale, an often-used scoring system in validated questionnaires [54,55]. However, this resulted in less detailed information about the emotional state of the participants; although an increase from 1 to 3 may seem significant, it simply indicates that the participant selected “(3) neutral/do not know,” which does not directly reflect an improvement. To encounter this, a visual analog scale of 0 to 10 or 0 to 100 might have been a better way to assess the outcomes in a more specific, sensitive way.

### Conclusions

This study has shown that DEEP seems a promising intervention for some, but not all, forensic psychiatric inpatients to practice deep breathing techniques in a novel and engaging way. DEEP is not a one-size-fits-all intervention that works for every patient, as the effectiveness in decreasing emotional tension, stress, and anger varied among the participants. Despite variation in quantitative results, both inpatients and the health care professionals found DEEP to be of added value in providing short-term relaxation. In addition, DEEP seems to be an engaging way to acquire deep breathing as a coping strategy. However, generalization to daily lives was difficult, which can be explained by problems with interoceptive awareness and lack of integration in treatment protocols. This underlines the need for a structural integration of interventions such as DEEP in existing treatment programs or psychoeducation. Future research should therefore focus not only on whether DEEP is effective, but also on why and for whom it is effective, by identifying patient-, context-, and intervention-related factors.

## Appendix

Multimedia Appendix 1 Overview of Single-Case Reporting Guideline in Behavioral Interventions (SCRIBE) checklist, linear regression models, and coding schemes.

## Abbreviations

ES: experience sampling
ESM: experience sampling method
LMM: linear mixed model
SCED: single-case experimental design
SCRIBE: Single-Case Reporting Guideline in Behavioral Interventions
VR: virtual reality

## Data Availability

The datasets generated and analyzed during this study are available from the corresponding author on reasonable request.
