# Supplementary material for: The Effect of the Virtual Reality–Based Biofeedback Intervention DEEP on Stress, Emotional Tension, and Anger in Forensic Psychiatric Inpatients: Mixed Methods Single-Case Experimental Design
Source: JMIR Form Res. 2025 Feb 12;9:e65206. doi: 10.2196/65206 (PMC11888111; doi:10.2196/65206)
Supplement: Multimedia Appendix 1 [file formative_v9i1e65206_app1.docx]

**Multimedia appendix 1:**

**Appendix 1:**

*SCRIBE Checklist for Single-Case Experimental Designs*

Tate, R.L., Perdices, M., Rosenkoetter, U., Shadish, W., Vohra, S., Barlow, D.H., Horner, R., Kazdin, A., Kratochwill, T., McDonald, S., Sampson, M., Shamseer, L., Togher, L., Albin, R., Backman, C., Douglas, J., Evans, J. J., Gast, D., Manolov, R., Mitchell, G., Nickels, L., Nikles, J., Ownsworth, T., Rose, M., Schmid, C. H. & Wilson, B. (2016). The Single-Case Reporting Guideline In BEhavioural Interventions (SCRIBE) Remedial and Special Education, 37(6): 370–380. doi:10.1177/0741932516652893

| Section | Item | Description | Page |
| --- | --- | --- | --- |
| Title and Abstract | Title | Clearly state the study uses a single-case experimental design. | 1 |
|  | Abstract | Summarize the study, including background, methods, results, and conclusions. | 2 |
| Introduction | Background | Describe the rationale, theoretical framework, and context for the study. | 3-5 |
|  | Research Questions/Hypotheses | Clearly state the objectives and/or hypotheses. | 6 |
| Methods | Participant Characteristics | Provide details such as age, gender, diagnosis, and relevant characteristics. | 6-7 |
|  | Setting | Describe the environment where the intervention was delivered. | 6-7 |
|  | Materials | Describe any materials, tools, or equipment used. | 7-8 |
|  | Independent Variable | Define and describe the intervention in detail, including components and procedures. | 10-11 |
|  | Dependent Variable | Describe the outcome measures, including operational definitions and measurement techniques. | 10-11 |
|  | Experimental Design | Specify the design type (e.g., ABAB, multiple baseline, alternating treatments). | 6 |
|  | Procedures | Outline the procedures, including baseline, intervention, and follow-up phases. | 9-10 |
|  | Fidelity of Implementation | Report how the consistency of the intervention delivery was ensured and assessed. | 11-12 |
| Results | Data Presentation | Present raw data in tables or graphs with clear labeling of phases. | 13-21 |
|  | Data Analysis | Describe methods for analyzing the data (e.g., visual analysis, statistical methods). | 10-12 |
|  | Effect Sizes (if applicable) | Report and interpret effect sizes, if calculated. | 13-21 |
| Discussion | Summary of Findings | Summarize key findings in relation to the research questions. | 21-22 |
|  | Interpretation | Discuss findings within the context of existing literature and theory. | 22-23 |
|  | Limitations | Address limitations related to design, implementation, and generalizability. | 24-25 |
|  | Implications | Discuss implications for practice, policy, or further research. | 22-23 + 25 |
| Additional Information | Funding and Conflicts of Interest | Declare funding sources and any potential conflicts of interest. | 26-27 |
|  | Ethical Approval | Report ethical approval or consent procedures where applicable. | 12 |
|  | Informed Consent | Indicate whether informed consent was obtained from participants or guardians. | 9 + 12 |

**Appendix 2**

*Individual linear regression models for emotional tension*

| Participant | Parameter | B | SE | t | *p* |
| --- | --- | --- | --- | --- | --- |
| P1 | Intercept | 3.965 | 0.088 | 45.024 | 0 |
|  | Time | -0.002 | 0.003 | -0.646 | 0.523 |
|  | DEEP | 0.035 | 0.312 | 0.111 | 0.912 |
|  | Time x DEEP | 0.002 | 0.011 | 0.184 | 0.855 |
| P2 | Intercept | 2.49 | 0.206 | 12.077 | 0 |
|  | Time | -0.006 | 0.006 | -1.07 | 0.29 |
|  | DEEP | -6.49 | 3.781 | -1.716 | 0.093 |
|  | Time x DEEP | 0.188 | 0.097 | 1.936 | 0.059 |
| P3 | Intercept | 1.985 | 0.127 | 15.607 | 0 |
|  | Time | 0.002 | 0.005 | 0.381 | 0.706 |
|  | DEEP | 1.875 | 0.459 | 4.081 | <0,001** |
|  | Time x DEEP | -0.053 | 0.016 | -3.4 | 0.002** |
| P4 | Intercept | 2.265 | 0.246 | 9.224 | 0 |
|  | Time | 0 | 0.007 | -0.04 | 0.968 |
|  | DEEP | -0.265 | 1.081 | -0.245 | 0.808 |
|  | Time x DEEP | 0 | 0.068 | 0.004 | 0.997 |
| P5 | Intercept | 2.802 | 0.157 | 17.859 | 0 |
|  | Time | -0.012 | 0.004 | -2.839 | 0.006** |
|  | DEEP | -0.802 | 1.72 | -0.466 | 0.643 |
|  | Time x DEEP | 0.012 | 0.067 | 0.186 | 0.853 |
| P6 | Intercept | 3.265 | 0.261 | 12.53 | 0 |
|  | Time | -0.009 | 0.007 | -1.251 | 0.216 |
|  | DEEP | -1.223 | 1.044 | -1.172 | 0.246 |
|  | Time x DEEP | 0.018 | 0.036 | 0.485 | 0.63 |

*P = <0,05

**p=<0,01

**Appendix 3**

*Individual linear regression models for stress*

| Participant | Parameter | B | SE | t | *p* |
| --- | --- | --- | --- | --- | --- |
| P1 | Intercept | 3.961 | 0.081 | 49.164 | 0 |
|  | Time | 0.002 | 0.003 | 0.567 | 0.574 |
|  | DEEP | -0.865 | 0.286 | -3.025 | 0.005** |
|  | Time x DEEP | 0.023 | 0.01 | 2.409 | 0.021* |
| P2 | Intercept | 2.425 | 0.206 | 11.794 | 0 |
|  | Time | -0.005 | 0.006 | -0.821 | 0.416 |
|  | DEEP | -6.425 | 3.77 | -1.704 | 0.095 |
|  | Time x DEEP | 0.187 | 0.097 | 1.925 | 0.061 |
| P3 | Intercept | 2.204 | 0.151 | 14.581 | 0 |
|  | Time | -0.006 | 0.006 | -0.964 | 0.341 |
|  | DEEP | -0.204 | 0.546 | -0.374 | 0.71 |
|  | Time x DEEP | 0.006 | 0.019 | 0.3 | 0.766 |
| P4 | Intercept | 1.998 | 0.166 | 12.047 | 0 |
|  | Time | 0.004 | 0.004 | 0.786 | 0.436 |
|  | DEEP | 0.002 | 0.73 | 0.003 | 0.997 |
|  | Time x DEEP | -0.004 | 0.046 | -0.077 | 0.939 |
| P5 | Intercept | 3.034 | 0.179 | 16.913 | 0 |
|  | Time | -0.014 | 0.005 | -2.821 | 0.007** |
|  | DEEP | 1.55 | 1.966 | 0.788 | 0.434 |
|  | Time x DEEP | -0.069 | 0.076 | -0.908 | 0.368 |
| P6 | Intercept | 3.506 | 0.248 | 14.127 | 0 |
|  | Time | -0.029 | 0.007 | -4.03 | <0,001** |
|  | DEEP | -0.631 | 0.994 | -0.634 | 0.528 |
|  | Time x DEEP | 0.004 | 0.035 | 0.103 | 0.919 |

*P = <0,05

**p=<0,01

**Appendix 4**

*Individual linear regression models for anger*

| Participant | Parameter | B | SE | t | *p* |
| --- | --- | --- | --- | --- | --- |
| P1 | Intercept | 3.405 | 0.198 | 17.179 | 0 |
|  | Time | -0.001 | 0.007 | -0.093 | 0.927 |
|  | DEEP | 0.455 | 0.703 | 0.647 | 0.522 |
|  | Time x DEEP | -0.032 | 0.024 | -1.348 | 0.186 |
| P2 | Intercept | 2.453 | 0.257 | 9.555 | 0 |
|  | Time | -0.002 | 0.008 | -0.284 | 0.778 |
|  | DEEP | -6.453 | 4.707 | -1.371 | 0.177 |
|  | Time x DEEP | 0.184 | 0.121 | 1.519 | 0.136 |
| P3 | Intercept | 2.07 | 0.102 | 20.296 | 0 |
|  | Time | -0.001 | 0.004 | -0.192 | 0.849 |
|  | DEEP | -0.07 | 0.368 | -0.189 | 0.851 |
|  | Time x DEEP | 0.001 | 0.013 | 0.06 | 0.953 |
| P4 | Intercept | 2.112 | 0.255 | 8.297 | 0 |
|  | Time | 0.004 | 0.007 | 0.52 | 0.606 |
|  | DEEP | -0.112 | 1.121 | -0.1 | 0.921 |
|  | Time x DEEP | -0.004 | 0.07 | -0.051 | 0.96 |
| P5 | Intercept | 2.349 | 0.104 | 22.63 | 0 |
|  | Time | -0.007 | 0.003 | -2.424 | 0.019** |
|  | DEEP | -0.349 | 1.138 | -0.307 | 0.76 |
|  | Time x DEEP | 0.007 | 0.044 | 0.158 | 0.875 |
| P6 | Intercept | 1.557 | 0.306 | 5.095 | 0 |
|  | Time | 0.009 | 0.009 | 0.981 | 0.331 |
|  | DEEP | 0.776 | 1.225 | 0.634 | 0.529 |
|  | Time x DEEP | -0.042 | 0.043 | -0.981 | 0.331 |

*P = <0,05

**p=<0,01

**Appendix 5**

*Coding schemes of interviews with patients (n=6) and therapists (n=5)*

**Table A**

*Experienced short- term effect of DEEP after session according to participants, with the (sub-)codes based on the data of the interviews with patients (n=6) and their therapists (n=5)*

| Codes and subcodes | Definition | Total*^a^* | Patients*^b^* | Therapists*^c^* |
| --- | --- | --- | --- | --- |
| **Emotional tension** | The extent to which DEEP has contributed to the short-term reduction of emotional tension after a session. |  |  |  |
| *Effect* | | 27 | 6(20) | 3(7) |
| *No effect* | |  |  | 1(1) |
| **Stress** | The extent to which DEEP has contributed to the short-term reduction of stress after a session. |  |  |  |
| *Effect* | | 12 | 5(7) | 3(5) |
| *No effect* | | 2 | 1(1) | 1(1) |
| **Anger** | The extent to which DEEP has contributed to the short-term reduction of anger after a session. |  |  |  |
| *Effect* | | 5 | 3(5) |  |
| *No effect* | | 5 | 2(4) | 1(1) |
| **Emotion regulation** | The extent to which DEEP has contributed to the short-term improvement of emotion regulation after a session. |  |  |  |
| *Effect* | |  |  |  |
| *No effect* | | 1 |  | 1(1) |

*^a^The total number of times a code was mentioned in all interviews. ^b^The number of patients that mentioned a code, and (#) the number of times the code was found in all interviews with patients. ^c^The number of therapists that mentioned a code, and (#) the total number of times the code was found in all interviews with therapists.*

**Table B**

*Experienced long-term effect of DEEP during the 20 days-SCED according to participants, with the (sub-)codes based on the data of the interviews with patients (n=6) and their therapists (n=5)*

| Codes and subcodes | Definition | Total*^a^* | Patients*^b^* | Therapists*^c^* |
| --- | --- | --- | --- | --- |
| **Emotional tension** | The extent to which DEEP has contributed to the reduction of emotional tension during the SCED of 20 days. |  |  |  |
| *Effect* | | 10 | 6(9) | 1(1) |
| *No effect* | | 2 |  | 2(2) |
| **Stress** | The extent to which DEEP has contributed to the reduction of stress during the SCED of 20 days. |  |  |  |
| *Effect* | | 6 | 3(4) | 1(2) |
| *No effect* | | 3 | 3(3) |  |
| **Anger** | The extent to which DEEP has contributed to the reduction of anger during the SCED of 20 days. |  |  |  |
| *Effect* | | 8 | 1(2) | 4(6) |
| *No effect* | | 7 | 5(6) | 1(1) |
| **Emotion regulation** | The extent to which DEEP has contributed to the improvement of emotion regulation during the SCED of 20 days. |  |  |  |
| *Effect* | | 4 |  | 3(4) |
| *No effect* | |  |  |  |

*^a^The total number of times a code was mentioned in all interviews. ^b^The number of patients that mentioned a code, and (#) the number of times the code was found in all interviews with patients. ^c^The number of therapists that mentioned a code, and (#) the total number of times the code was found in all interviews with therapists.*
